# Supplementary material for: Progression of prostate cancer reprograms MYC-mediated lipid metabolism via lysine methyltransferase 2A
Source: Discov Oncol. 2022 Oct 1;13:97. doi: 10.1007/s12672-022-00565-3 (PMC9526773; doi:10.1007/s12672-022-00565-3)
Supplement: Supplementary file 3 — (PDF 1537 KB) [file 12672_2022_565_MOESM3_ESM.pdf]

Supplementary Materials for

Progression of prostate cancer reprograms MYC-mediated lipid metabolism via lysine methyltransferase 2A

by

Nichelle C. Whitlock et al.

Supplementary Methods
Supplementary Table Legends
Supplementary Figures
Supplementary References

Supplementary Methods

Microarray analysis of laser capture microdissected prostate tumor foci

Affymetrix Human Exon Array data of 26 laser capture microdissected human prostate tumors of Gleason patterns 3 and 4 (1) were downloaded from GEO using accession number GSE52560. SCAN.UPC (2) normalized intensity values were used.

RNA-seq analysis of TCGA tumors

Whole transcriptomes of The Cancer Genome Atlas prostate adenocarcinoma (TCGA-PRAD) primary prostate cancer cohort (3) were retrieved from the NCI Genomics Data Commons (https://gdc.cancer.gov) via access to dbGaP phs000178. Cases with Gleason scores of 3+5=8 and 5+3+8 were excluded. Sample reads were aligned and processed as with the primary LCM RNA-seq cohort.

Supplementary Table Legends

Supplementary Table 1. Ingenuity upstream regulator candidates based on gene body or transcriptional start site loci for anti-AR or anti-HOXB13 ChIP-seq. For each potential upstream regulator, its rank from either core or redistributed sites is shown along with the *P* value of overlap with the canonical IPA gene set for that regulator.

Supplementary Table 2. Differentially enriched genes corresponding to AR- or HOXB13-bound sites (peaks) based on *KMT2A* expression.

Supplementary Figures

Supplementary Figure 1

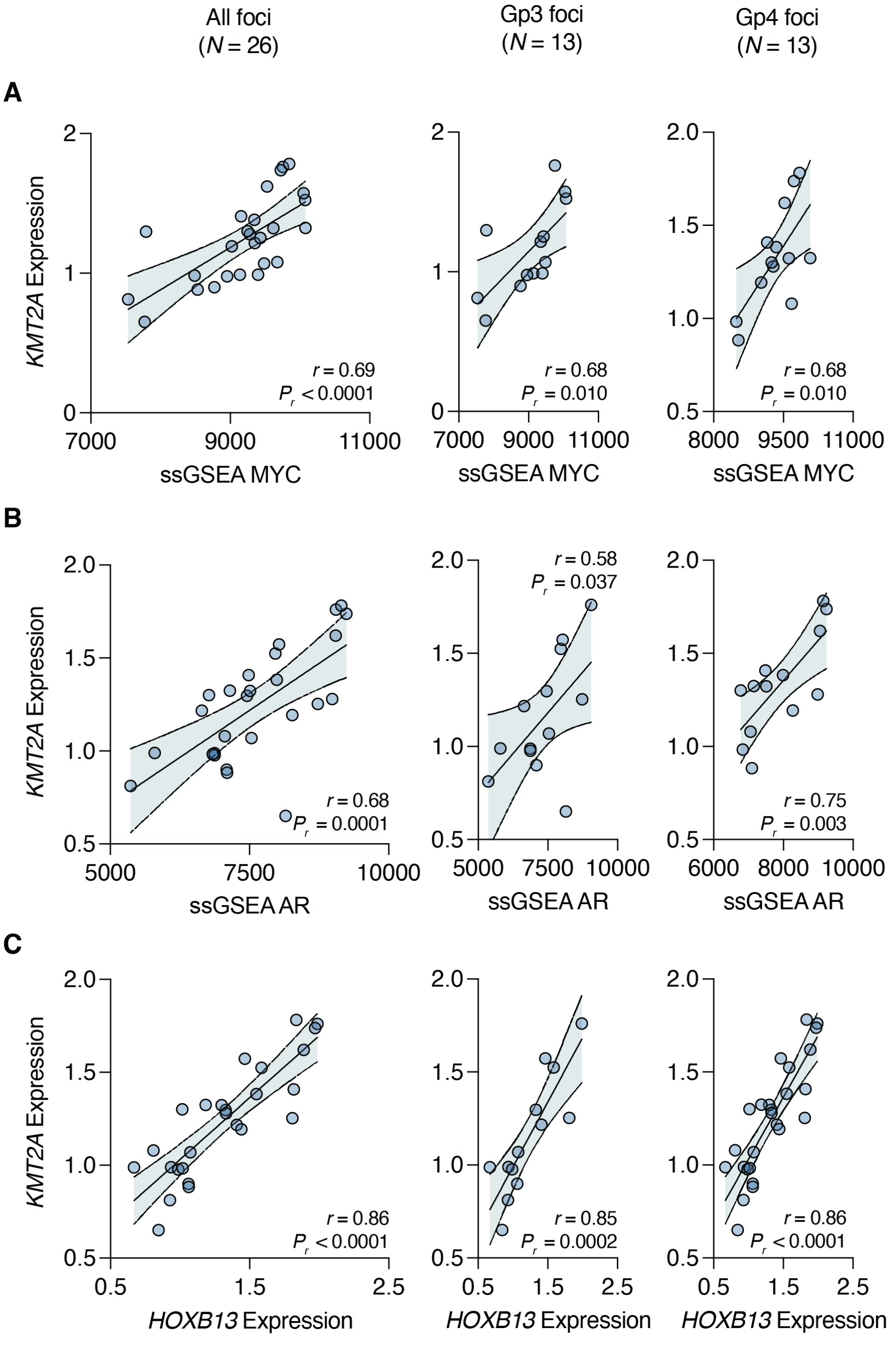

Supplementary Figure 1. Association of *KMT2A* expression with prostate cancer drivers in primary disease. A, B, C. Pearson correlation of the normalized expression of *KMT2A* with a 54-gene ssGSEA MYC activity score (A), a 266-gene ssGSEA AR activity score (B), or the normalized expression of *HOXB13* (C) a cohort of 26 laser capture microdissected foci of human prostate tumors). The error bars represent the 95% confidence bands for linear regression. For each correlation, the foci are subdivided into Gp3 (left), Gp4 (right, including intraductal tumors).

Supplementary Figure 2

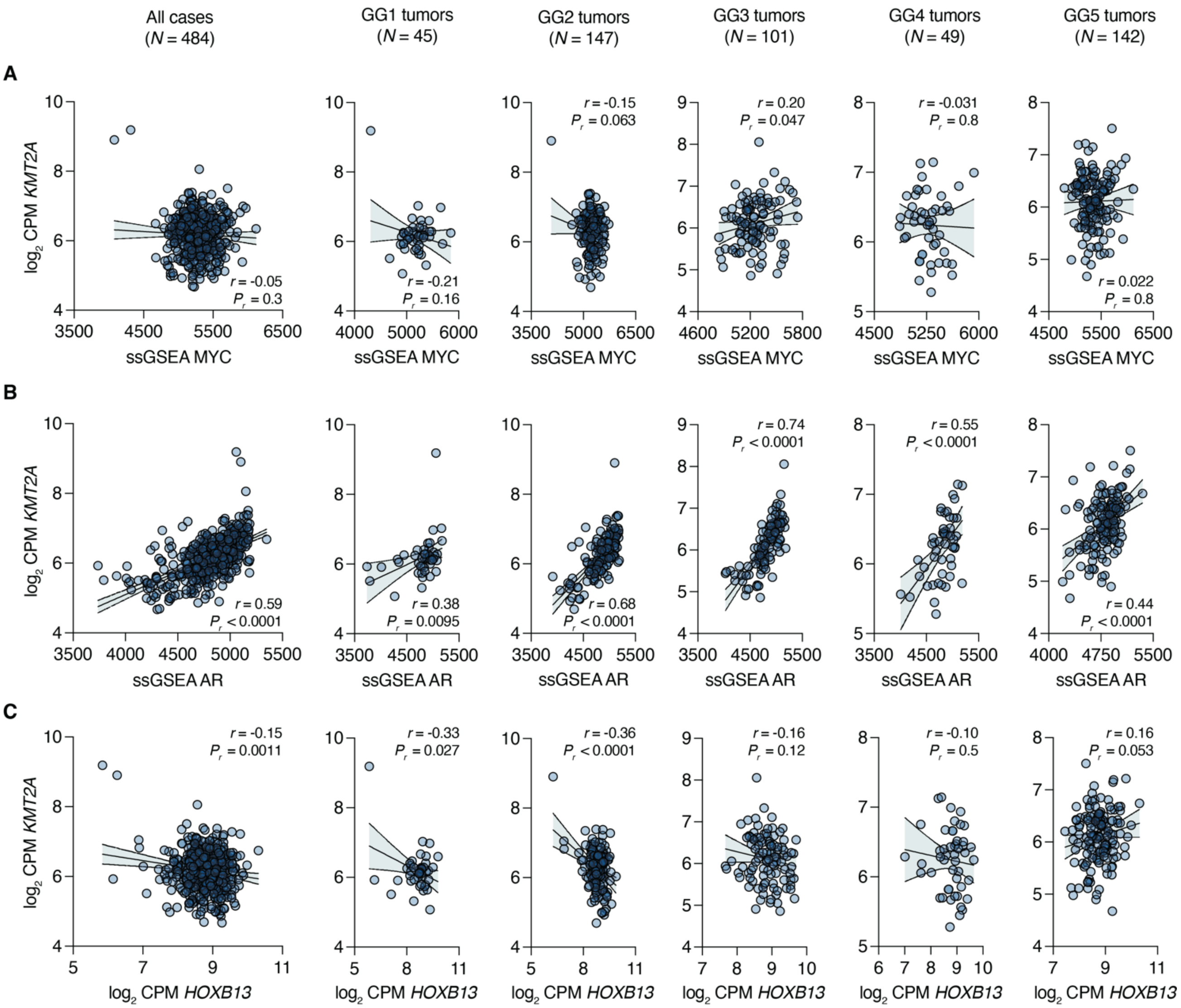

Supplementary Figure 2. Association of *KMT2A* expression with prostate cancer drivers in primary disease. A, B, C. Pearson correlation of the $\log_2$ CPM expression level for *KMT2A* with a 54-gene ssGSEA MYC activity score (A), a 266-gene ssGSEA AR activity score (B), or the $\log_2$ CPM expression level for *HOXB13* (C) a cohort of 484 human prostate tumors from the prostate cancer TCGA (TCGA-PRAD). The error bars represent the 95% confidence bands for linear regression. For each correlation, the foci are subdivided into Gleason ISUP Grade Groupings (1-5); tumors scored Gleason 3+5=8 or 5+3=8 excluded.

Supplementary Figure 3

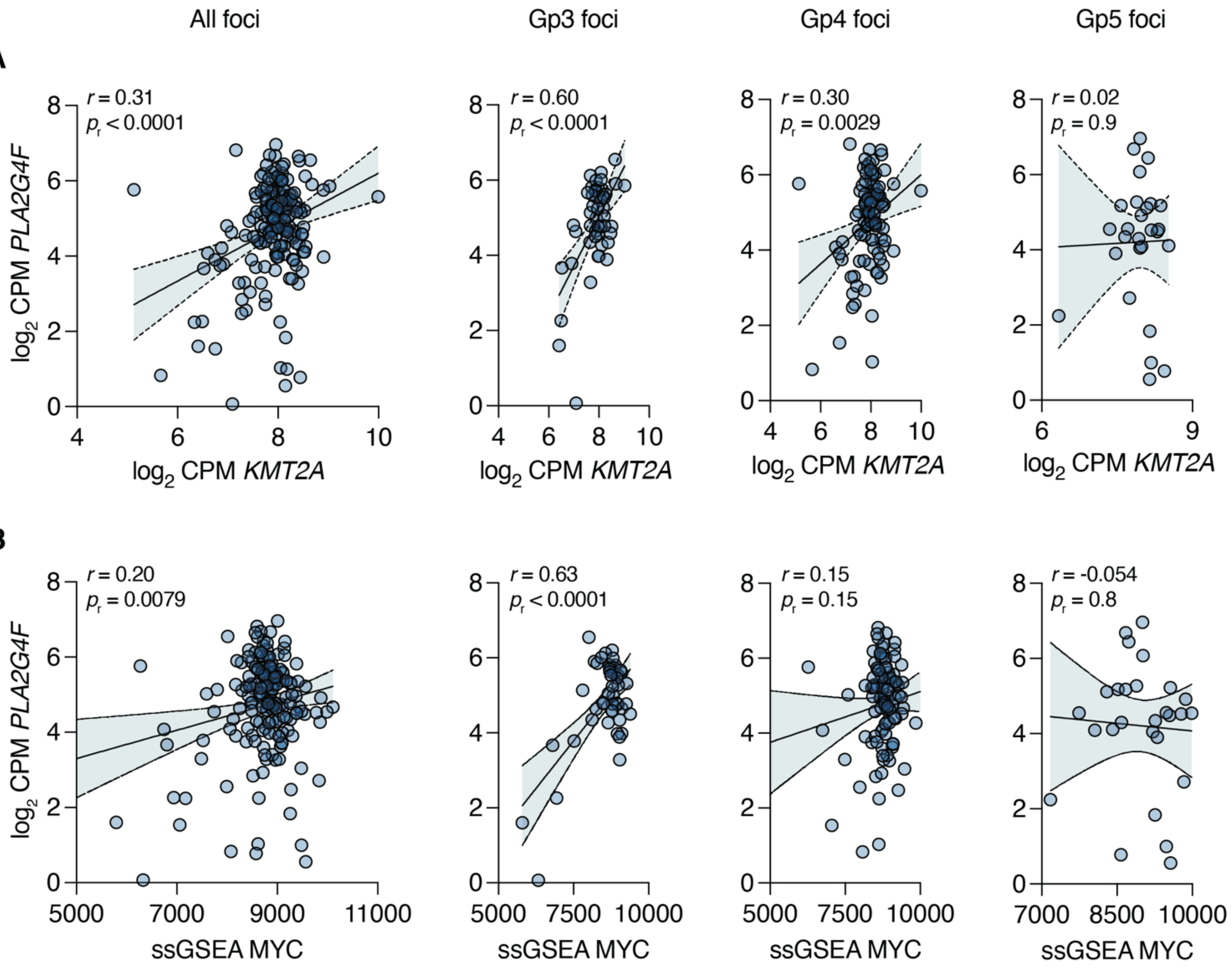

Supplementary Figure 3. Decreasing association between *PLA2G4F* and *KMT2A* expression and MYC activity in primary prostate cancer. A, B. Pearson correlation of the $\log_2$ CPM expression level of *PLA2G4F* with $\log_2$ CPM expression level of *KMT2A* (A) or the 54-gene ssGSEA MYC activity score (B) in a cohort of laser capture microdissected foci of human prostate tumors. The error bars represent the 95% confidence bands for linear regression. Samples with $\log_2$ CPM *PLA2G4F* < 0 were excluded. For each correlation, the foci are subdivided into Gp3 (left), Gp4 (middle, including intraductal tumors), and Gp5 (right).

Supplementary References

1. Sowalsky AG, Kissick HT, Gerrin SJ, Schaefer RJ, Xia Z, Russo JW*, et al.* Gleason Score 7 Prostate Cancers Emerge through Branched Evolution of Clonal Gleason Pattern 3 and 4. Clin Cancer Res **2017**;23:3823-33
2. Piccolo SR, Sun Y, Campbell JD, Lenburg ME, Bild AH, Johnson WE. A single-sample microarray normalization method to facilitate personalized-medicine workflows. Genomics **2012**;100:337-44
3. Cancer Genome Atlas Research N. The Molecular Taxonomy of Primary Prostate Cancer. Cell **2015**;163:1011-25
